# BMSC affinity peptide-functionalized β-tricalcium phosphate scaffolds promoting repair of osteonecrosis of the femoral head

**DOI:** 10.1186/s13018-019-1243-5

**Published:** 2019-07-04

**Authors:** Guozong Wang, Yi Li, Tiantong Sun, Congcong Wang, Li Qiao, Yi Wang, Kangkang Dong, Tao Yuan, Jiazheng Chen, Guanqiao Chen, Shui Sun

**Affiliations:** 1 Department of Joint Surgery, Shandong Provincial Hospital Affiliated to Shandong University, 324 Jingwuweiqi Road, Jinan, 250021 Shandong China; 2 College of Clinical Medicine, Shandong University, Jinan, 250012 Shandong China; 3 Shandong First Medical University & Shandong Academy of Medical Sciences, Taian, 271016 Shandong China

**Keywords:** Osteonecrosis of the femoral head, Bone marrow-derived mesenchymal stem cell, Affinity peptide, β-tricalcium phosphate

## Abstract

**Background:**

Osteonecrosis of the femoral head (ONFH) is a disabling disease. Early treatment is crucial to the prognosis of the disease. Core decompression (CD) is one of the most commonly used methods for the treatment of early ONFH. But it could not prevent the collapse of the necrotic femoral head. How to improve the therapeutic effect of early ONFH on the basis of CD has become an area of focused research.

**Methods:**

Functional β-tricalcium phosphate (β-TCP) scaffolds modified by DPIYALSWSGMA (DPI) peptide, a bone marrow-derived mesenchymal stem cell (BMSC) affinity peptide, were constructed using an adsorption/freeze-drying strategy. The affinity of DPI peptide towards rabbit BMSCs was investigated using flow cytometry and fluorescence cytochemistry. In vitro cell adhesion assay was performed to study the adherent ability of rabbit BMSCs on functional β-TCP scaffolds. After the rabbit model of early ONFH was established, DPI peptide-modified and pure β-TCP scaffolds were transplanted into the remaining cavity after CD. Meanwhile, rabbits treated with pure CD were used as blank control. Twelve weeks after surgery, histological analysis was performed to show the therapeutic effect of three methods on early ONFH.

**Results:**

The result of ImageXpress Micro Confocal indicated that fabricated DPI peptide-modified functional β-TCP scaffolds exhibited green fluorescence. In flow cytometry, the average fluorescence intensity for rabbit BMSCs incubated with FITC-DPI was significantly higher than that of FITC-LSP (*P* = 2.733 × 10^−8^). In fluorescence cytochemistry, strong fluorescent signals were observed in rabbit BMSCs incubated with FITC-DPI and FITC-RGD, whereas no fluorescent signals in cells incubated with FITC-LSP. In cell adhesion assay, the number of adherent cells to β-TCP-DPI scaffolds was more than that of pure β-TCP scaffolds (*P* = 0.033). The CD + β-TCP-DPI group expressed the lowest vacant bone lacunae percentage compared to CD group (*P* = 2.350 × 10^−4^) and CD + β-TCP group (*P* = 0.020). The expression content of COL1 in CD + β-TCP-DPI group was much higher than CD group (*P* = 1.262 × 10^−7^) and CD + β-TCP group (*P* = 1.666 × 10^−7^) according to the integrated optical density (IOD) analyses.

**Conclusion:**

Functional β-TCP scaffolds modified by DPI peptide were successfully synthesized using an adsorption/freeze-drying strategy. DPI peptide has good affinity towards rabbit BMSCs. The adhesion of rabbit BMSCs on DPI peptide-modified β-TCP scaffolds was apparently enhanced. CD followed by implantation of DPI peptide-modified β-TCP scaffolds can apparently improve the treatment of early ONFH compared with pure CD and CD followed by implantation of unmodified β-TCP scaffolds. Our current study provides an improved method for the treatment of early ONFH.

## Introduction

Osteonecrosis of the femoral head (ONFH) is a disabling disease affecting relatively young population [1]. This progressive disease can lead to collapse of articular surface of the femoral head and eventual osteoarthritis of the hip joint [2]. It is estimated that 20,000–30,000 new patients are diagnosed annually in the USA [3]. The treatment of ONFH is still challenging and the effect is not completely satisfactory. The ultimate goal for the treatment of ONFH is hip preservation [4]. If ONFH cannot be cured effectively in the early stage, it will lead to collapse of the femoral head and the patients need to be treated with total hip arthroplasty (THA) [5]. Because patients are relative young, choice of THA means that these patients need to undergo arthroplasty more than once. Core decompression (CD) is one of the most commonly used methods for the treatment of early ONFH [6]. Although it can significantly relieve pain, it does not halt progression of the disease [7]. How to improve the therapeutic effect of early ONFH on the basis of CD has become an area of focused research. It has been realized that the support to the necrotic femoral head is further reduced because of the remaining cavity after CD, which may lead to the collapse of the femoral head more easily [7]. So bone, tantalum rods and lots of bone-graft substitutes have been filled into the bone tunnels of CD in order to improve the effect of pure CD in the treatment of ONFH [8–10].

Tricalcium phosphate (TCP) and hydroxyapatite (HA) are two members of calcium phosphate family, which are commonly used in orthopedics as bone grafts [11]. The calcium/phosphorus ratio of TCP is 1.5 and that of HA is 1.67. HA is hardly adsorbed in vivo, which blocks the formation of new bone [12]. β-TCP is one of the existing forms of TCP, which depends on the method of manufacture and sintering. β-TCP is a totally adsorbable bioceramic. As a calcium bone-graft substitute, it has been studied in the treatment of ONFH combined with CD [13]. However, it was reported that the imbalance between the faster degradation of β-TCP and the weaker ability of osteogenesis led to the collapse of the femoral head [14]. Moreover, β-TCP lacks biological stimulatory activity [15]. These make β-TCP not an ideal biomaterial to be applied in the treatment of early ONFH combined with CD.

In order to change or improve the properties of materials, processing and modification is a feasible method. For example, zinc oxide (ZnO) nanoparticles were added to the nanocrystalline hydroxyapatite (n-HA) scaffolds coated by gelatin-ibuprofen to play a role of the anti-infection effect [16]. Surface modification is a commonly used and effective method. It has been reported that the surface of demineralized bone matrix particles was modified by bone marrow-derived mesenchymal stem cell (BMSC) affinity peptide to promote cartilage regeneration [17]. As we know, BMSCs also play an important role in the repair of ONFH because of their ability of osteogenesis and secretion of cytokines such as vascular endothelial growth factor (VEGF) to promote vascular regeneration [18]. DPIYALSWSGMA (DPI) peptide, discovered by phage display, is a linear polypeptide with specific affinity towards BMSCs [19]. The surface of β-TCP could be modified with DPI peptide to fabricate functional scaffold which has the property to recruit BMSCs [20]. So in our current study, we constructed functional β-TCP scaffolds modified by DPI peptide and investigated the therapeutic effect of CD combined with implantation of the functional β-TCP scaffolds on the treatment of early ONFH. The purpose of this study was to provide a new idea for clinical treatment of early ONFH.

## Materials and methods

### Outline of the study

**Fig. 1:**
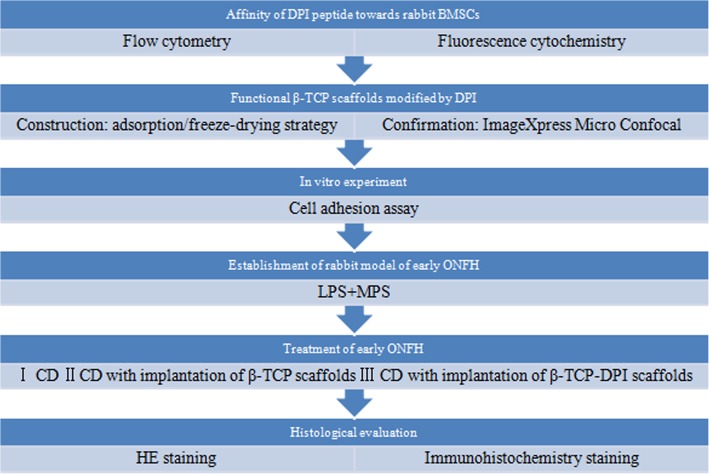
Flow chart of the study

The affinity of DPI peptide towards rabbit BMSCs was investigated using flow cytometry and fluorescence cytochemistry. Then we constructed functional β-TCP scaffolds modified by DPI peptide using an adsorption/freeze-drying strategy. In vitro cell adhesion assay was performed to investigate the effect of the functional β-TCP scaffolds on adhesion of rabbit BMSCs. After the establishment of the rabbit model of early ONFH, the therapeutic effect of CD combined with implantation of functional β-TCP scaffolds on early ONFH was studied (Fig. 1).

### Cell culture

**Fig. 2:**
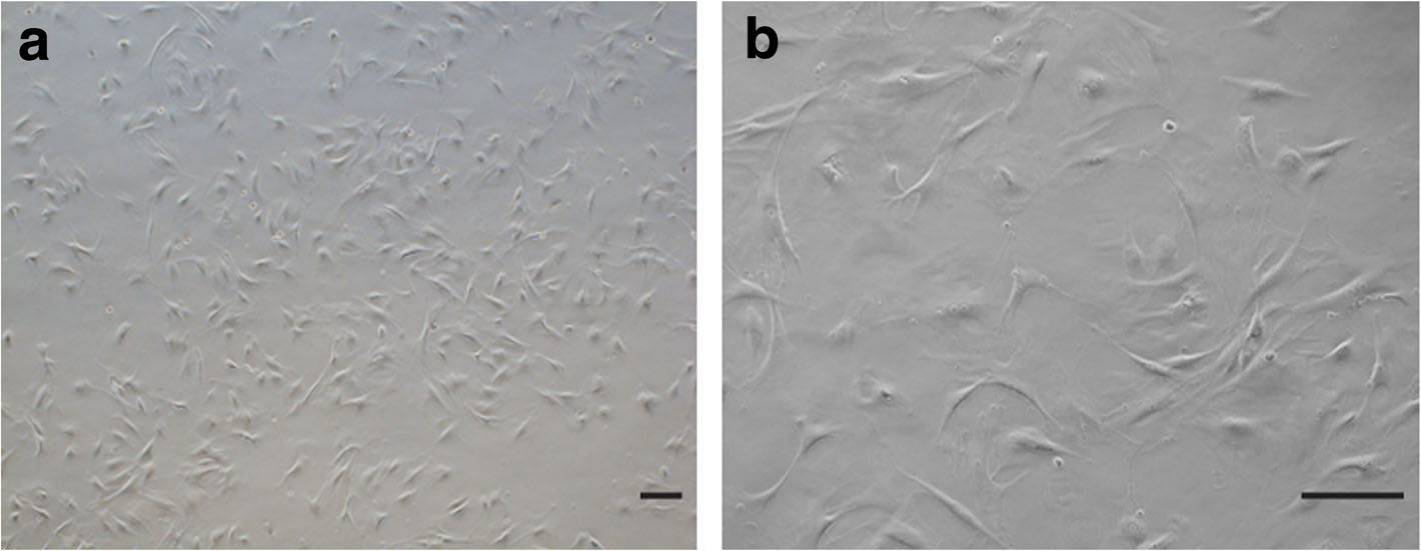
The rabbit BMSCs showed a spindle-shaped or fibroblastic appearance—**a** magnification, × 40, and **b** magnification, × 100; scale bar, 250 μm

Rabbit BMSCs (cat. no. RBXMX-01001) were purchased from Cyagen Biosciences, Inc. (Santa Clara, CA, USA) (Fig. 2). Cells were cultured in low glucose Dulbecco’s modified Eagle’s medium (DMEM; cat. no. 01-051-1A; Biological Industries, Ltd., Kibbutz Beit-Haemek, Israel) with L-glutamine containing 10% fetal bovine serum (cat. no. 10099141; Gibco; Thermo Fisher Scientific, Inc., Waltham, MA, USA) and antibiotics (100 U/ml penicillin and 0.1 mg/ml streptomycin; cat. no. 15140122; Gibco; Thermo Fisher Scientific, Inc.) in an incubator with 5% CO_2_ at 37 °C. Media was changed every 2–3 days. Cells were passaged when they reached 80–90% confluence.

### Synthesis of peptides

In addition to DPI, a peptide (LSPSAGAYIDWM) with the same chain length as DPI, but scrambled, was used as the negative control and designated as LSP. RGD (arginine-glycine-aspartic acid) was used as the positive control. DPI, LSP, and RGD were synthesized through solid-phase peptide synthesis using 9-fluorenylmethoxycarbonyl chemistry (Scilight-Peptide Inc., Beijing, China). An aminohexanoic acid was conjugated to the amino terminal of all peptides to facilitate fluorescein-5-isothiocyanate (FITC) labeling. All peptides were stored in − 20 °C.

### Affinity assay of DPI peptide towards rabbit BMSCs via flow cytometry

The rabbit BMSCs were washed twice with PBS (cat. no. 02-024-1A; Biological Industries, Ltd.) and dissociated with 0.25% trypsin-EDTA (cat. no. 25200-056; Gibco; Thermo Fisher Scientific, Inc.). The cell suspension was centrifuged at 250×*g* for 5 min at room temperature to collect cell sedimentation. The cells were further washed twice with PBS and then incubated with 10 μM FITC-labeled peptides dissolved in PBS for 1 h at 37 °C to allow cell binding. The affinity of the peptides towards rabbit BMSCs was analyzed quantitatively through flow cytometer (FCM; BD LSR Fortessa; Becton, Dickinson and Company, Franklin Lakes, NJ, USA) at a wavelength of 488 nm and FlowJo 7.6.1 software (Tree Star, Inc., Ashland, OR, USA).

### Affinity assay of DPI peptide towards rabbit BMSCs via fluorescence cytochemistry

The rabbit BMSCs were cultured in 24-well dishes containing coverslips until 70–90% confluence was achieved. The cells were washed twice with PBS, fixed in 4% paraformaldehyde, permeabilized with 0.5% Triton X-100, and blocked with goat serum. Then the cells were incubated with 100 μM FITC-labeled peptides dissolved in PBS for 1 h at 37 °C. The cells were incubated with 80 nM rhodamine phalloidin (cat. no. CA1610; Beijing Solarbio Science & Technology Co., Ltd., Beijing, China) for 30 min at room temperature to show the cytoskeleton. The nuclei were counterstained with 10 μg/ml DAPI (cat. no. C0065; Beijing Solarbio Science & Technology Co., Ltd.) for 10 min at room temperature. The cells on the round coverslips were observed under a fluorescence microscope (type BX63; Olympus Corporation, Tokyo, Japan).

### Synthesis of DPI peptide-modified β-TCP (β-TCP-DPI) scaffolds

Disk-shaped β-TCP bioceramic (diameter, 6 mm; height, 2 mm) (Fig. 4a) and cylindric β-TCP bioceramic (diameter, 4 mm; height, 14 mm) (Fig. 4b) were purchased from Shanghai Bio-lu Biomaterials Co., Ltd. (Shanghai, China). The porosity was 75% ± 10% by mercury intrusion porosimetry (MIP). DPI peptide was adsorbed onto β-TCP through an adsorption/freeze-drying strategy according to a previously reported protocol [20]. Briefly, β-TCP was incubated for 24 h in peptide solution containing 100 μg/ml DPI in PBS in a ratio of 1.0 g β-TCP to 2.0 ml solution. Incubation was carried out at room temperature with gentle shaking to ensure equilibration of the peptide with all exposed surfaces of the porous β-TCP. Unadsorbed peptide was removed by washing five times in PBS with gently shaking over a 24 h period. The β-TCP-DPI scaffolds were dried in vacuo for 1 h and stored at − 20 °C in moisture-proof containers. FITC-labeled DPI peptide-modified β-TCP scaffolds were fabricated and examined using ImageXpress Micro Confocal (Molecular Devices, LLC, Sunnyvale, CA, USA).

### Cell adhesion assay

Cell Counting Kit-8 (CCK-8; cat. no. 96992; Sigma-Aldrich; Merck KGaA, Darmstadt, Germany) reagent was used to evaluate cell adhesion on the scaffolds according to a previously reported protocol [21]. All scaffolds were sterilized through ultraviolet (UV) light exposure in a super clean bench before usage. The rabbit BMSCs were washed twice with PBS and dissociated with 0.25% trypsin-EDTA. The cell suspension was centrifuged at 250×*g* for 5 min at room temperature to collect cell sedimentation. Cells were resuspended for a specified concentration (6 × 10^4^ cells/ml) in serum-free DMEM. Then 200 μl of prepared cell suspension was added into 96-well dishes containing disk-shaped β-TCP or β-TCP-DPI scaffolds. After 3 h of incubation, the scaffolds were removed to new wells and washed three times sufficiently with PBS. Then 100 μl of fresh medium and 10 μl of CCK-8 reagent were added. The cells adhering to scaffolds were incubated for another 4 h at 37 °C. The absorbance at 450 nm was measured by an automated microplate reader (type 1510; Thermo Fisher Scientific, Inc.). All procedures were repeated at least three times.

### Treatment of early ONFH using β-TCP-DPI scaffolds

#### Experimental animals

Twenty-four healthy male New Zealand rabbits with body weight of 2–3 kg (mean 2.65 kg) were used. The rabbits received a standard laboratory diet and water ad libitum. The present study was performed in compliance with Guide for the Care and Use of Laboratory Animals of the National Institutes of Health. The experimental protocol was approved by the Experimental Animal Ethics Committee of Shandong Provincial Hospital Affiliated to Shandong University (Shandong, China).

#### Establishment of the rabbit model of early ONFH

The model of early ONFH was established according to a previously reported protocol [22]. Briefly, the rabbits were intravenously injected with 10 μg/kg body weight of lipopolysaccharide (LPS; cat. no. G5032; Servicebio, co., Ltd., Wuhan, Hubei, China). Then, 24 h later, three injections of 20 mg/kg body weight of methylprednisolone (MPS; Pfizer, Inc., New York City, NY, USA) were given intramuscularly at a time interval of 24 h. Six weeks after injection of MPS, ONFH was confirmed by histological examination. The femoral heads of rabbits randomly selected were excised, fixed in 4% paraformaldehyde, decalcified in 0.5 M EDTA, and embedded in paraffin. Then tissue blocks were cut into 4-μm-thick sections along the coronal plane, stained with hematoxylin and eosin (H&E) and observed under light microscope (type BX63; Olympus Corporation).

#### Animal surgery procedure

Eighteen rabbits of early ONFH were divided into three groups, one group for the treatment of pure CD, one group for the treatment of CD followed by implantation of unmodified β-TCP scaffolds, and one group for the treatment of CD followed by implantation of β-TCP-DPI scaffolds. All scaffolds were sterilized by γ-irradiation before surgery. The animals were anesthetized by ear vein injection of 3% Pentobarbital sodium (1 ml/kg body weight; Sigma-Aldrich; Merck KGaA). Then the animals were operated according to a previously reported technique [23]. Briefly, a standard lateral approach to expose the greater trochanter was made under aseptic conditions. CD was performed using a 4-mm surgical drill and a 4 × 14-mm bone tunnel from the greater trochanter to the femoral head was created. Cylindric unmodified β-TCP scaffolds and β-TCP-DPI scaffolds were placed into the bone tunnels. The wound was closed layer by layer.

#### Histological evaluation

At 12 weeks post-surgery, rabbits were anesthetized and sacrificed. Histomorphology of rabbit femoral head was measured. Briefly, the proximal femurs containing the femoral head were excised, fixed in 4% paraformaldehyde, decalcified in 0.5 M EDTA, and embedded in paraffin. Then tissue blocks were cut into 4-μm-thick sections along the coronal plane, stained with H&E, and observed under light microscope (type BX63; Olympus Corporation). To assess changes in bone necrosis, the rate of vacant bone lacunae of the femoral head was calculated. Immunohistochemistry (IHC) staining of sections of the femoral head was also performed with mouse anti-type I collagen (COL1) antibody (1:2000, cat. no. GTX26308; GeneTex, Inc., Irvine, CA, USA) to assess the expression content of COL1. Images were obtained using a light microscope (type BX63; Olympus Corporation).

### Statistical analyses

In our experiment, data was expressed as mean ± standard deviation. In cell adhesion assay, Student’s *t* test was performed to compare two groups. In flow cytometry and histological analysis, one-way analysis of variance followed by Dunnett’s *t* test was performed for comparison of three groups. The significant level was set as *P* < 0.05. SPSS 24.0 (IBM Corp., Armonk, NY, USA) software was used for data analysis.

## Results

### Peptide-affinity assay towards rabbit BMSCs

**Fig. 3:**
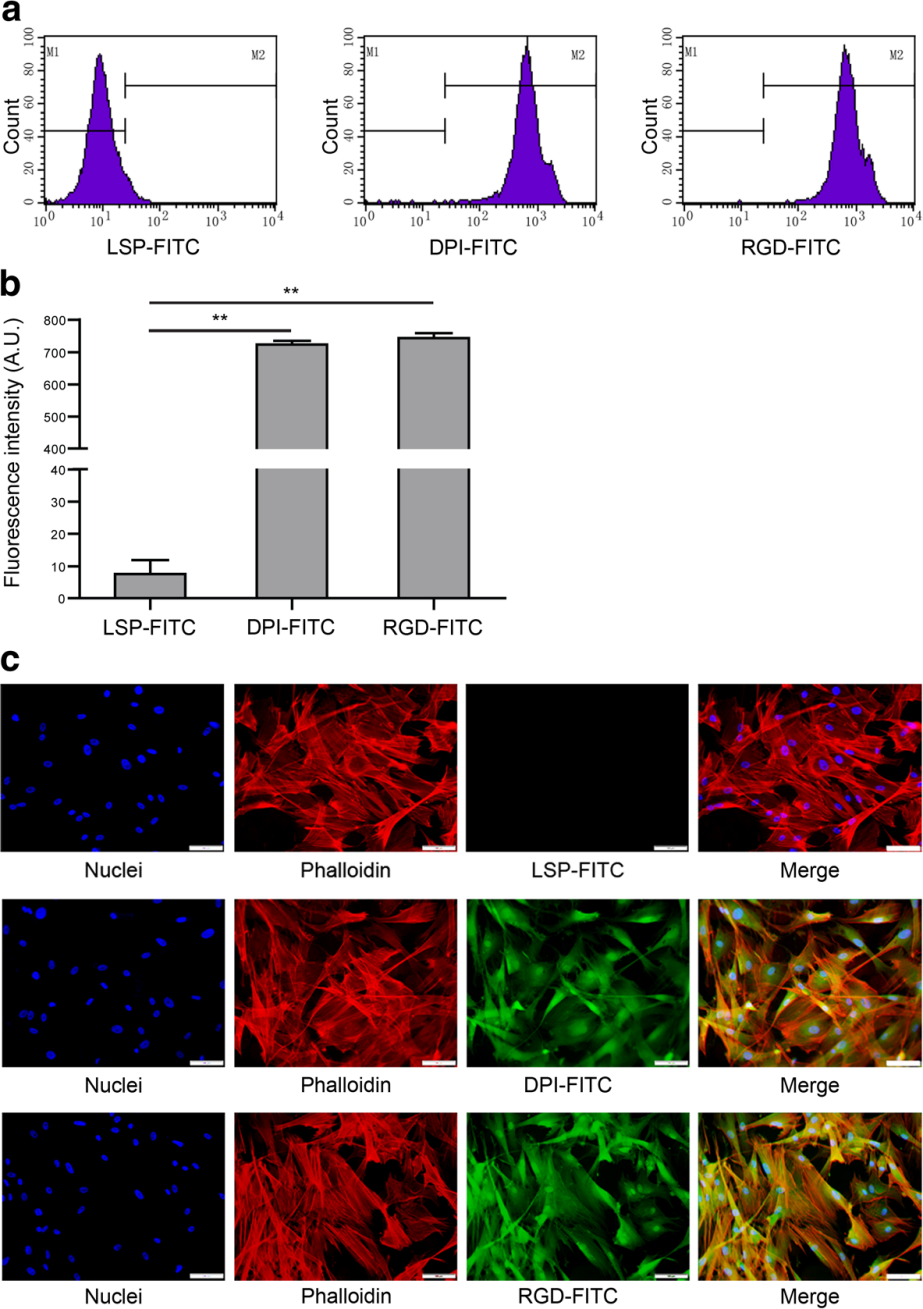
The rabbit BMSCs were incubated with FITC-labeled LSP, DPI, or RGD and analyzed by FCM. **a** The peak of the experimental group (DPI) and positive control group (RGD) shifted to the right markedly compared with the negative control group (LSP). **b** The average fluorescence intensity for the cells incubated with FITC-DPI was higher than that of FITC-LSP (*n* = 3; *P* = 2.733 × 10^−8^). **c** The cells incubated with FITC-LSP, FITC-DPI, or FITC-RGD were observed under a fluorescence microscope. Strong FITC signals were observed in cells incubated with FITC-DPI and FITC-RGD, whereas no FITC signals were observed in cells incubated with FITC-LSP. The nuclei were stained with DAPI. The cytoskeleton was stained with rhodamine phalloidin; scale bar, 100 μm

Rabbit BMSCs were incubated for 1 h with FITC-labeled LSP, DPI, or RGD and measured via FCM. The peak of the experimental group (DPI) and positive control group (RGD) shifted to the right markedly compared with the negative control group (LSP) (Fig. 3a). The average fluorescence intensity for the cells incubated with FITC-DPI was higher than that of FITC-LSP and the difference was statistically significant (*P* = 2.733 × 10^−8^; Fig. 3b). Rabbit BMSCs were also incubated with FITC-labeled LSP, DPI, or RGD and observed under a fluorescence microscope. Strong fluorescent signals were observed in the cells incubated with FITC-DPI and FITC-RGD, whereas no fluorescent signals were observed in the cells incubated with FITC-LSP (Fig. 3c). These results suggested that DPI peptide has high affinity towards rabbit BMSCs.

### Synthesis of β-TCP-DPI scaffolds

**Fig. 4:**
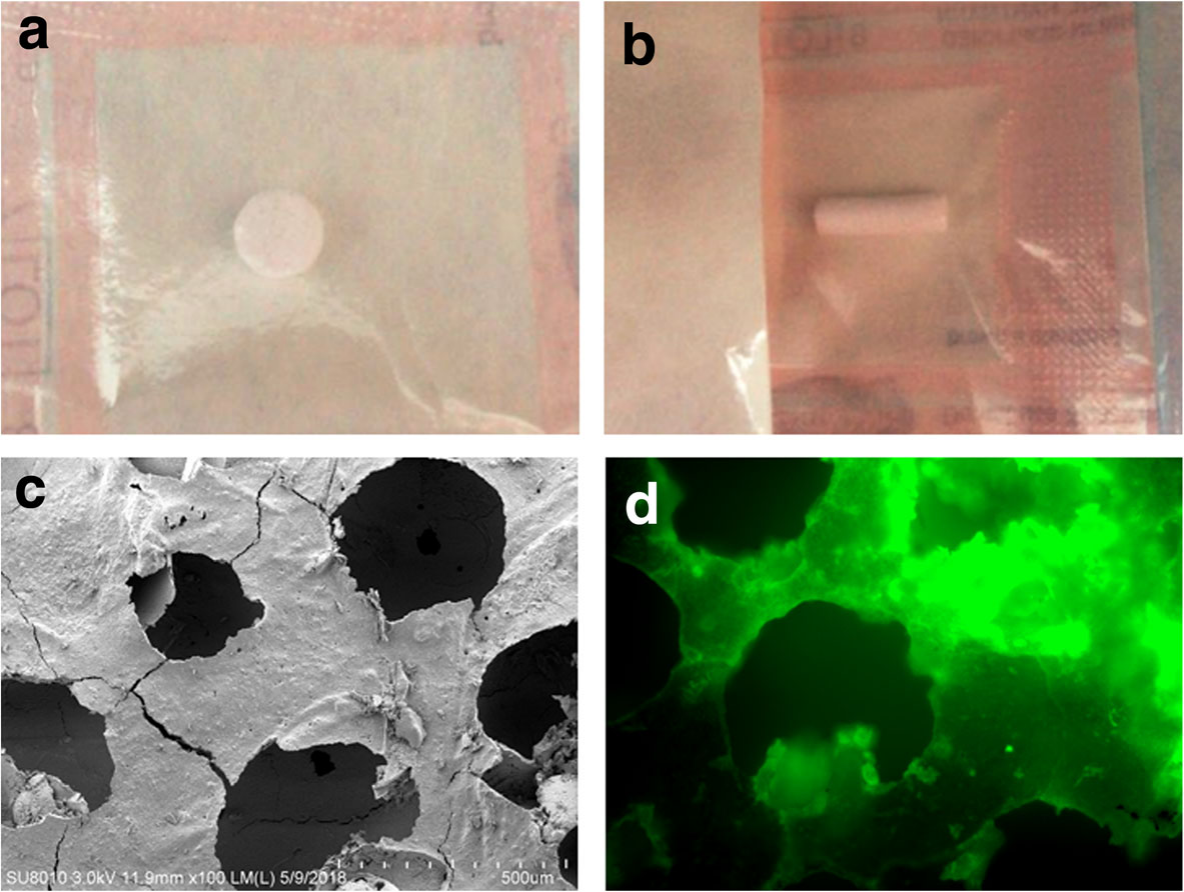
**a** disk-shaped and **b** cylindric β-TCP scaffold is a kind of porous bioceramic. **c** Scanning electron microscope (SEM) image of disk-shaped β-TCP bioceramic. **d** The FITC-DPI peptide-modified β-TCP scaffolds using an adsorption/freeze-drying strategy exhibited green fluorescence

The DPI peptide was adsorbed onto β-TCP for surface modification using an adsorption/freeze-drying strategy. After operation, the β-TCP scaffolds exhibited green fluorescence (Fig. 4d). This phenomenon indicated successful adsorption of DPI peptide onto the surface of β-TCP because the DPI peptide was pre-labeled with FITC. This result proved that we successfully constructed functional β-TCP composite materials modified by BMSC affinity peptide DPI.

### The adhesion of BMSCs to β-TCP-DPI scaffolds was apparently enhanced compared with pure β-TCP scaffolds

**Fig. 5:**
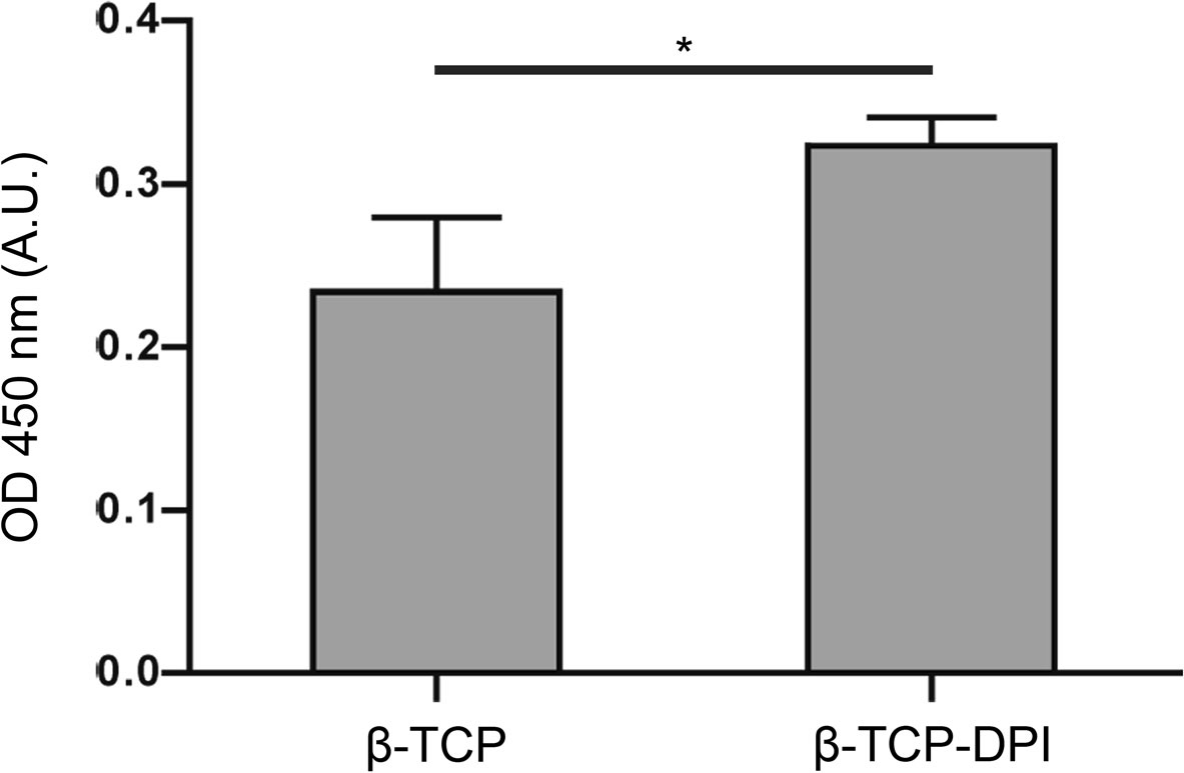
Cell adhesion of rabbit BMSCs was measured by Cell Counting Kit-8 assay 3 h after cell suspension was added into 96-well plates containing β-TCP-DPI or pure β-TCP scaffolds. The average OD value of the former was higher than the latter, suggesting that the number of adherent cells on β-TCP-DPI scaffolds was more than that of pure β-TCP scaffolds. The result indicated that the adhesion of rabbit BMSCs to β-TCP-DPI scaffolds was enhanced compared with pure β-TCP scaffolds. Data was expressed as mean ± standard deviation; *n* = 3; *P* = 0.033

In cell adhesion assay, cell suspension of rabbit BMSCs was added into 96-well dishes containing β-TCP-DPI or pure β-TCP scaffolds and incubated for 3 h for cell adhesion. The average optical density (OD) value of the former was higher than the latter and the difference was statistically significant (*P* = 0.033; Fig. 5), suggesting that the number of adherent cells to β-TCP-DPI scaffolds was more than that of pure β-TCP scaffolds.

### Model evaluation and treatment of core decompression with implantation of scaffolds

**Fig. 6:**
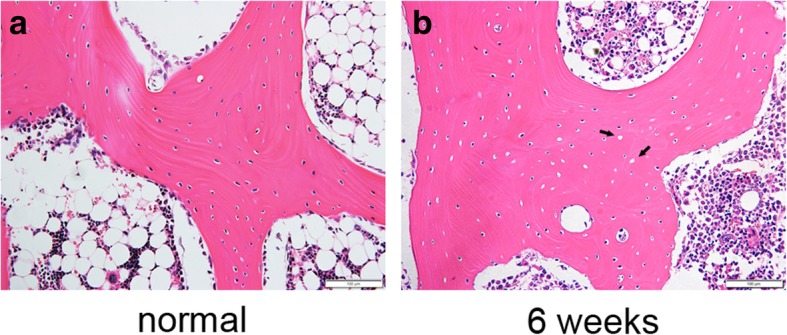
The rabbit model of early ONFH was established by combination of LPS and MPS. Six weeks after injection of MPS, the femoral heads of rabbits randomly selected were made into paraffin sections and stained with hematoxylin and eosin. A large number of vacant bone lacunae could be seen in the necrotic femoral head (**b**) compared with normal femoral head (**a**); magnification, × 200; scale bar, 100 μm

Osteonecrotic lesions with typical features were found in photomicrographs of H&E-stained sections. A large number of vacant bone lacunae could be seen in the bone matrix of necrotic femoral head compared with normal femoral head (Fig. 6).

**Fig. 7:**
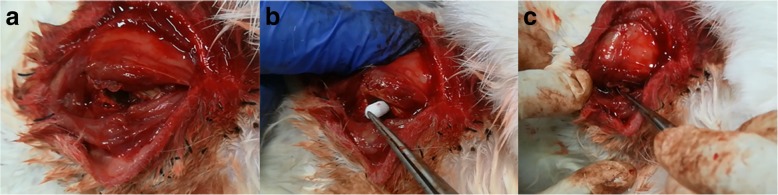
The procedure of CD and implantation of scaffolds. **a** A 4 × 14 mm bone tunnel was created by a 4-mm surgical drill from the greater trochanter to the femoral head. **b** β-TCP or β-TCP-DPI scaffolds were placed into the bone tunnels, respectively. **c** The implanted scaffold was compacted

Figure 7 demonstrated the surgical procedure of CD with implantation of scaffolds. In addition to the pure CD group, the unmodified or DPI peptide-modified β-TCP scaffolds were placed into the bone tunnels. No rabbits showed infection after surgery.

### Histological evaluation

**Fig. 8:**
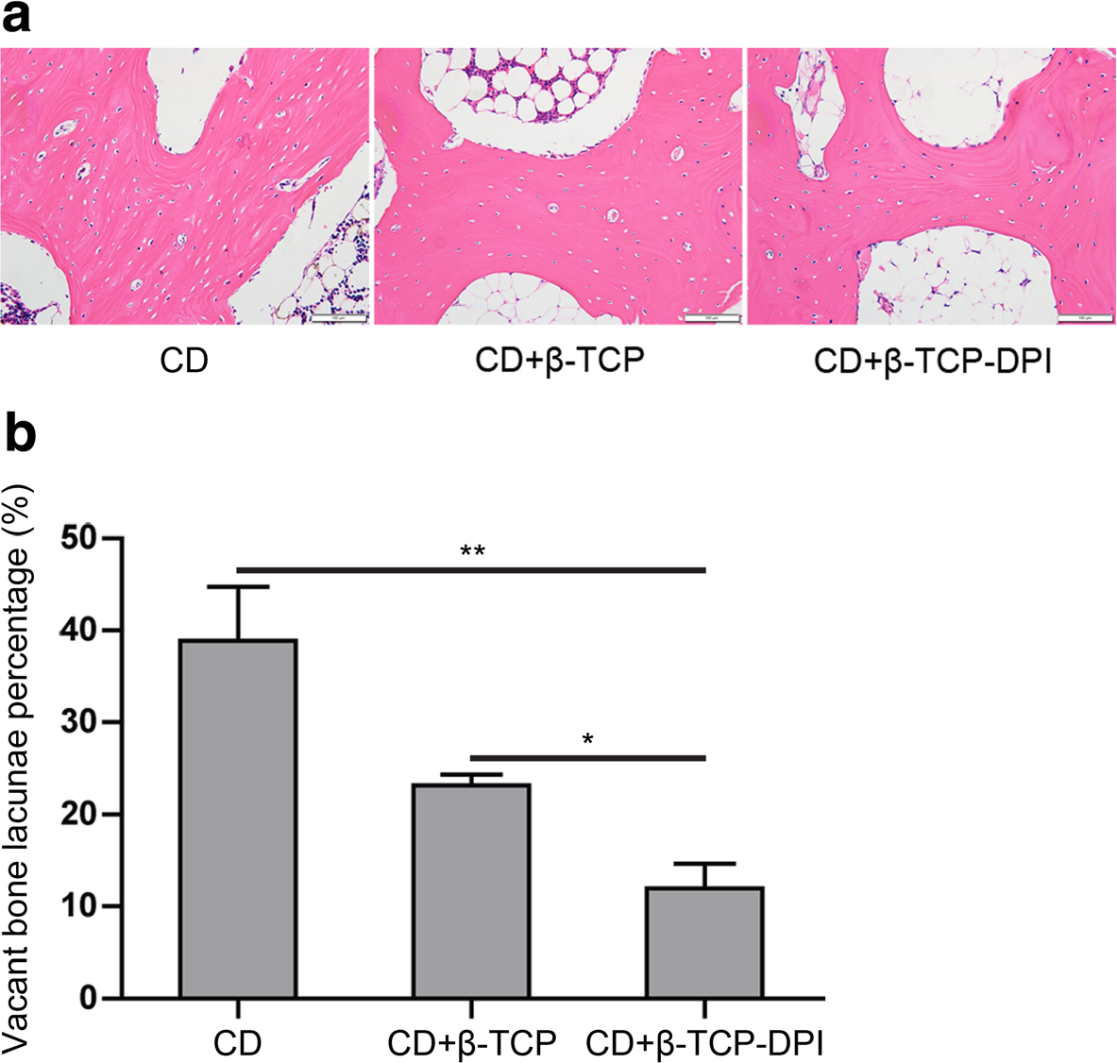
**a** Vacant bone lacunae among the trabecular matrix of femoral heads were seen in three groups. **b** CD + β-TCP-DPI group expressed the lowest vacant bone lacunae percentage compared to CD group (*P* = 2.350 × 10^−4^) and CD + β-TCP group (*P* = 0.020). This result indicated that the therapeutic effect of CD combined with implantation of β-TCP-DPI scaffolds was superior to CD and CD combined with implantation of unmodified β-TCP scaffolds. Data was expressed as mean ± standard deviation

**Fig. 9:**
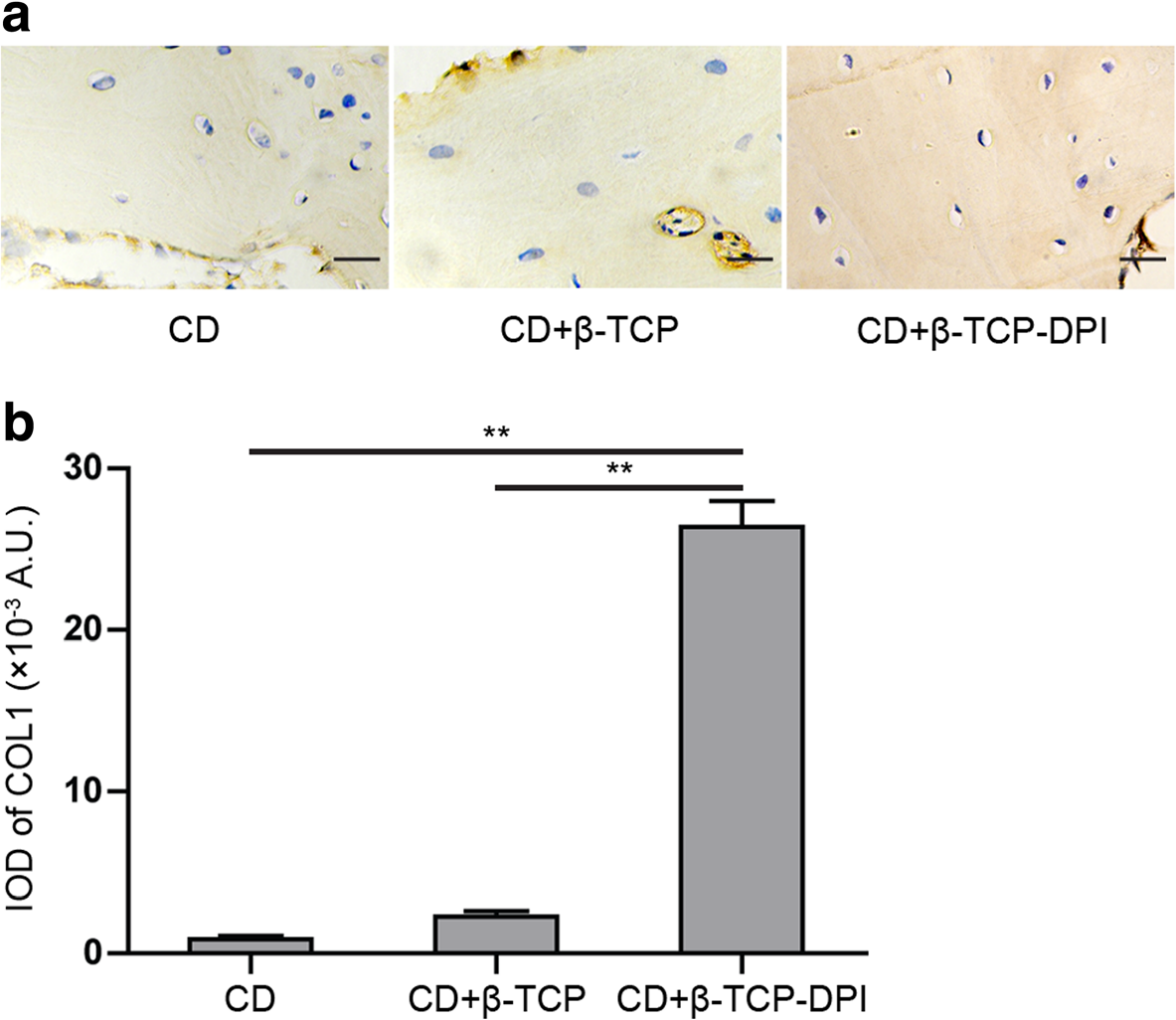
**a** The result of IHC staining indicated positive COL1 expression in all groups. The content of COL1 in CD + β-TCP-DPI group was higher than the other two groups; magnification, × 400; scale bar, 50 μm. **b** The IOD of CD + β-TCP-DPI group was higher than CD group (*P* = 1.262 × 10^−7^) and CD + β-TCP group (*P* = 1.666 × 10^−7^). This result indicated that the therapeutic effect of CD combined with implantation of β-TCP-DPI scaffolds was superior to CD and CD combined with implantation of unmodified β-TCP scaffolds. Data was expressed as mean ± standard deviation

The rabbits were sacrificed at weeks 12 after surgery. The femoral heads were retrieved to assess the changes in bone necrosis by histological analysis. Vacant bone lacunae among the trabecular matrix of femoral heads were seen in three groups (Fig. 8a). The group of CD combined with implantation of β-TCP-DPI scaffolds expressed the lowest vacant bone lacunae percentage compared to the group of pure CD (*P* = 2.350 × 10^−4^) and the group of CD combined with implantation of β-TCP scaffolds (*P* = 0.020) (Fig. 8b). IHC staining of COL1 was analyzed using IPP 6.0 (Media Cybernetics, Inc., Rockville, MD, USA) software to assess the therapeutic effect of three groups. The result indicated positive COL1 expression in all groups (Fig. 9a). The content of COL1 in the group of CD combined with implantation of β-TCP-DPI scaffolds was much higher than the group of pure CD (*P* = 1.262 × 10^−7^) and the group of CD combined with implantation of β-TCP scaffolds (*P* = 1.666 × 10^−7^) according to the integrated optical density (IOD) analyses (Fig. 8b). These findings indicated that CD combined with implantation of β-TCP-DPI scaffolds has better effect on the treatment of early ONFH.

## Discussion

ONFH can lead to collapse of the femoral head and secondary osteoarthritis of the hip joint, and early diagnosis and treatment are crucial to the prognosis of the disease [24]. CD can reduce intraosseous pressure of the femoral head and relieve pain. It is the most common procedure for the treatment of early ONFH [25]. However, the therapeutic effect of this method has been controversial. It is believed that bone tunnels after CD cause loss of strong support to the femoral head and the femoral head is prone to collapse [7]. Bone, tantalum rods or bone-graft substitutes are now used for structural filling of cavities left from CD. Tantalum implants were supposed to potentially prevent collapse of the femoral head and promote repair of necrotic femoral head through structural support and osteoinductivity. But short-term results and histological analysis of retrieved tantalum implants during conversion to THA were not satisfactory [26]. As a bone graft material, bioceramic has attracted the attention of researchers in biomedical field [27, 28]. There is report of CD combined with HA composite for the treatment of ONFH [29]. But HA is hardly adsorbed in vivo, which blocks the formation of new bone [12]. “Advanced Core Decompression” (ACD) is a relatively new technique using a percutaneous expandable reamer to remove necrotic tissue from the femoral head and refilling the drill hole and the defect with composite calcium sulfate-calcium phosphate bone-graft substitute. However, collapse of the femoral head still appears because this bone graft is only osteoconductive but lacks osteoinductivity [30].

β-TCP is one of the commonly used bone-graft substitutes in orthopedics. It can be completely degraded and absorbed in vivo [31]. But its absorption is faster than the new bone formation [32]. Meanwhile, β-TCP lacks osteogenicity [33]. BMSCs have the potential of osteogenesis and angiogenesis [18]. BMSCs play an important role in the repair of ONFH. But the number of BMSCs in necrotic femoral head is less, which further reduces the ability of osteogenesis [34]. DPI is a polypeptide with specific affinity towards BMSCs, which was identified through phage display technology [19]. In our present study, we fabricated DPI peptide-modified β-TCP scaffolds to recruit BMSCs towards necrotic femoral head, make up for the deficiency of β-TCP, and improve the therapeutic effect of ONFH.

Artificial porous bone substitutes have been produced and studied as a possible bone replacement more and more widely [35]. β-TCP scaffold used in our study is a kind of porous bioceramic which is sintered at high temperature of 1100 °C. It is composed of completely interconnected spherical holes, which are beneficial to the growth of new bone tissue and blood vessels. Porous structure of β-TCP provides large surface area for cell attachment and adhesion.

As shown in Fig. 3, through flow cytometry and fluorescence cytochemistry assay, DPI peptide was confirmed to bind to rabbit BMSCs. Because the surface of β-TCP was modified with DPI peptide, the modified material was endowed with the ability of recruiting BMSCs. So in vitro experiment showed that the recruitment and adhesion of BMSCs onto the β-TCP-DPI scaffolds were apparently enhanced (Fig. 5). These provided the basis for our subsequent study on the treatment of rabbit early ONFH.

A rabbit model of early ONFH was induced by combination of LPS and MPS. It was reported that ONFH gradually developed 6 weeks after injection of MPS [36], which was similar to stage II of osteonecrosis (ON) clinically (Ficat and Arlet classification system) [37]. In the current study, 6 weeks after injection of MPS, the result of H&E staining showed that, compared with the normal femoral head, a large number of vacant bone lacunae appeared in the femoral head of modeling rabbit (Fig. 6). On the basis of the above reasons, we can infer that the model of early ONFH was successfully constructed.

Twelve weeks after surgery, the therapeutic effect of pure CD, CD combined with implantation of unmodified β-TCP scaffolds, and CD combined with implantation of β-TCP-DPI scaffolds on the treatment of early ONFH was investigated. The results of histological analysis indicated that the therapeutic effect of CD combined with implantation of β-TCP-DPI scaffolds was better. Although CD can reduce the intraosseous pressure of the femoral head and relieve pain of the hip, the mechanical support to the femoral head is weakened due to the residual cavity, which is more likely to lead to the collapse of the femoral head and affect the repair of the necrotic femoral head. It cannot halt progression of the disease [38]. In CD + β-TCP group, β-TCP scaffolds were filled into the bone tunnels to enhance the support to the femoral head. But due to the weak repair ability of necrotic femoral head, the osteogenic repair could not make up for the degradation of β-TCP scaffolds. This will also affect the repair of ONFH. In CD + β-TCP-DPI group, because osteogenesis and angiogenesis was elevated following the recruitment of BMSCs to the femoral head by the DPI peptide-modified β-TCP scaffolds, the repair ability of necrotic femoral head was enhanced and better therapeutic effect was obtained.

## Conclusion

In our study, we synthesized functional β-TCP scaffolds by adsorption of DPI peptide, a BMSC affinity peptide, onto the surface of β-TCP using an adsorption/freeze-drying strategy. The high affinity of DPI peptide towards rabbit BMSCs was further confirmed using flow cytometry and fluorescence cytochemistry. Adhesion of rabbit BMSCs on DPI peptide-modified β-TCP scaffolds was apparently enhanced. Using a rabbit model of early ONFH, functional β-TCP scaffolds were implanted into the bone tunnels of CD. Results demonstrated that the therapeutic effect of CD combined with implantation of functional β-TCP scaffolds on the treatment of early ONFH was superior to CD and CD combined with implantation of unmodified β-TCP scaffolds. Our current study provides a novel method for the treatment of early ONFH. Our study also offers a promising way to improve BMSC-based tissue engineering therapy. Because the DPI peptide-modified β-TCP scaffold has the function of recruiting BMSCs and promoting tissue repair, it may also become an appropriate alternative for the treatment of many challenging orthopedic diseases including fracture nonunion and osteochondral defects.

## Data Availability

The datasets used and analyzed during the current study are available from the corresponding author on reasonable request.

## Abbreviations

BMSC: Bone marrow-derived mesenchymal stem cell
CCK-8: Cell Counting Kit-8
CD: Core decompression
COL1: Type I collagen
DMEM: Dulbecco’s modified Eagle’s medium
FCM: Flow cytometer
FITC: Fluorescein-5-isothiocyanate
IHC: Immunohistochemistry
IOD: Integrated optical density
LPS: Lipopolysaccharide
MPS: Methylprednisolone
OD: Optical density
ONFH: Osteonecrosis of the femoral head
β-TCP: β-tricalcium phosphate
